# On a new species of *Micrambe* from Africa (Coleoptera, Cryptophagidae)

**DOI:** 10.3897/zookeys.748.23856

**Published:** 2018-03-04

**Authors:** José Carlos Otero, José Manuel Pereira

**Affiliations:** 1 Departamento de Zoología, Genética y Antropología Física, Facultad de Biología, 15782 Santiago de Compostela, Spain

**Keywords:** Cameroon, *Micrambe camerunensis* sp. n., new species, taxonomic key, taxonomy

## Abstract

A new species of *Micrambe* Thomson, 1863 (Coleoptera, Cryptophagidae), *Micrambe camerunensis* **sp. n.** from Cameroon is described and illustrated. No other record of any Cryptophagidae of Cameroon is known. The differential diagnosis is established in relation to a group of other species of the genus.

## Introduction

The African fauna of *Micrambe* is significantly rich in species, although it is scarcely known. Predictably, as the study of its fauna continues, the number of species will rise significantly. Coombs and Woodroffe (1962) suggest that according to the size of the aedeagus, the African species constitute a phylogenetic group different from the Palearctic ones. A large number of species in South Africa were examined and no significant differences were found in that character. On the contrary, more variability (in relation to the Palearctic and Oriental species) was noticed in some morphological characteristics (pubescence, size, and shape of the eyes, etc.) and they present some exclusive characters such as setae in the last abdominal ventrite, protuberance in the margins of the aedeagus, etc. (Otero 2012).

The aim of this account is to contribute to the knowledge of Cryptophagidae from Africa. The study of abundant material of the genus *Micrambe* (Coleoptera, Cryptophagidae) from different museums suggests that the knowledge of this family in Africa needs to be updated. The study of numerous specimens borrowed from BMNH has allowed us to describe the new species, *Micrambe camerunensis* sp. n.

## Methods

The terminology and the measurements of the new species follow Otero (2005, 2011, 2012, 2017). Structures were measured under a Leica M205C stereomicroscope equipped with an Application Suite analysis system. Acronyms: **L** – length; **WL** – width/length ratio; **E** – eccentricity of the eyes (width/half of the length). The width is measured across the widest part of a line joining the anterior and posterior limit of the eye. Length is the maximum length of the eye. **L** is used for length in dorsal view, **W** for width, and **Ø** for diameter.

**Institutional abbreviations**

**BMNH** British Museum of Natural History, London, United Kingdom;

**MHNG** Muséum d’Histoire Naturelle, Genève, Suisse (coll. Y. Gomy);

**MNHN** Museum National d’Histoire Naturelle, Paris, France;

**SMNS** Staatliches Museum fur Naturkunde, Stuttgart, Germany;

**MSNF** Museo di Storia Naturale, Firenze, Italy (coll. Bartolozzi);

**NHMW** Naturhistorisches Museum Wien, Vienna, Austria;

**RMCA** Royal Museum Central Africa, Tervuren, Belgium;

**TMSA** Transvaal Museum, Pretoria, South Africa.

## Taxonomy

### Micrambe camerunensis sp. n.

Taxon classificationAnimaliaColeopteraCryptophagidae

http://zoobank.org/AC2B8A96-51A9-4AEF-8410-C4E739202EF6

Figures 1–5

#### Material examined.

“Holotype m*. **CAMEROON**. Mt Cameroon, Ist. Plateau, 12.I.1932 /10.000–12.000 ft (Leg. M. Steele)/B.M. 1934-240 (placed in BMNH)”//“Paratype, 5 m*m* and 3 f*f*, same locality, date and legtor as Holotype”// “1 f*, Mt Cameroon, Highest Point/13,360 ft (leg. M. Steele), B.M. 1934-240”//”1 f*, Mt Cameroon, Mann’s Quelle/7,400 ft, 3.II.1932 (leg. M. Steele), B.M. 1934-240”.

#### Diagnosis.

Morphologically, *Micrambe camerunensis* is very similar to other *Micrambe* in many external features, but can be distinguished by the configuration of the male genital apparatus.

#### Description.

Length: 1.7–1.9 mm. Body oval, elongated and convex. Reddish grey-brown; appendages and first antennal articles yellowish grey-brown. Pubescence simple, short (L= 0.025–0.040 mm) and flattened. Metathoracic wings absent.

Transverse *head* (WL = 1.9–2.1). Punctation well -marked and dense; distance between punctures shorter than their diameter (Ø = 0.014–0.016 mm). Normal eyes (L = 0.127 mm), hemispherical or sub-hemispherical and protruding (E = 1.1–1.2). Eye facets smaller (Ø = 0.012 mm) than head punctures. Short antennae (Fig. 2) (L = 0.580 mm), not surpassing the base of the pronotum. 1^st^ antennomere spherical; 2^nd^ and 3^rd^ as long as 1^st^ but narrower; 4^th^ and 6^th^ 1.8 times shorter than 3^rd^; 5^th^ 1.3 times longer than 4^th^; 7^th^ sub-squared and 1.1 times as long as 6^th^; 8^th^ transverse and as long as 7^th^; 9^th^ and 10^th^ equally long and strongly transverse; 11^th^ elongated.

**Figures 1–5.**
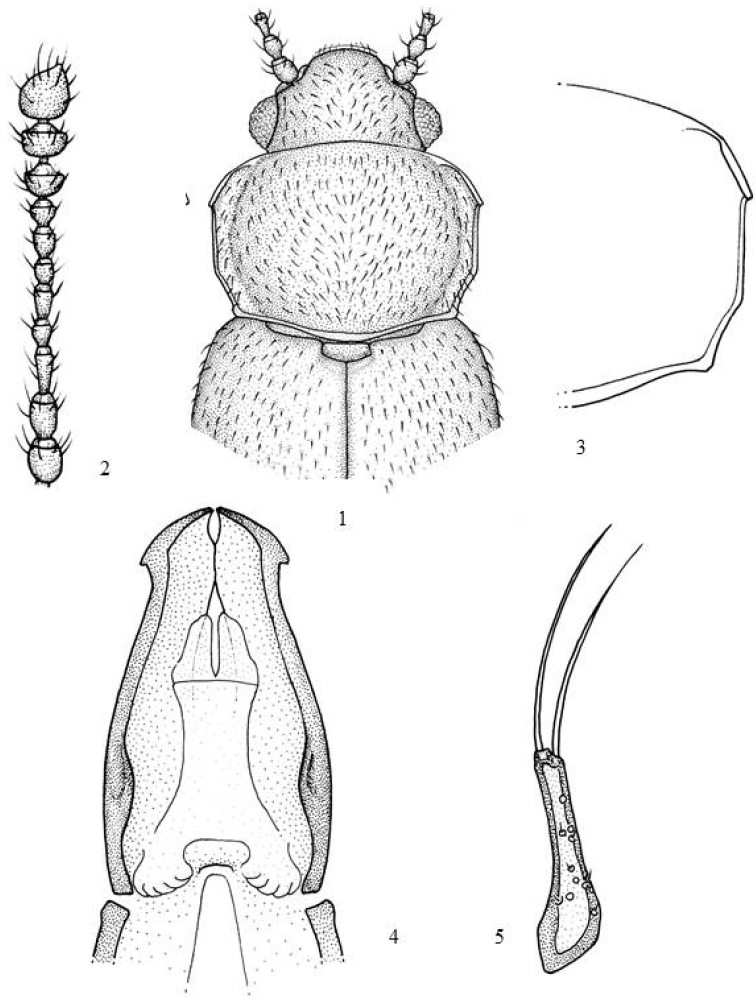
*Micrambe camerunensis*: **1** General view **2** antenna **3** pronotum **4** aedeagus **5** paramere.

*Pronotum* (Figs 1, 3) slightly transverse (WL = 1.5). Callosity oval, elongated, large (1/4 times as long as side); not surpassing the lateral margin of the pronotum. Callosity margin strong. Callosity side not visible from above. Gland pore present but not visible. Callosity not angled rearwards but forming a 38.33°–39° angle with the body axis. Lateral margins parallel from the callosity to the basal quarter and converging from there to the base. Posterior angles obtuse. Basal groove reduced. Basal foveae not visible. Punctation well -marked and dense; distance between punctures shorter than their diameter (Ø = 0.016–0.018 mm).

*Elytra* three times as long and 1.2 times as wide as pronotum. Punctation more dispersed than on pronotum; distance between punctures greater than their diameter (Ø = 0.016–0.018 mm).

*Mesosternum* with a narrow medial area, strongly concave, with sides slightly lifted, curved and converging towards a weakly emarginate apex.

*Tarsal formula* 5-5-5 in males and 5-5-5 in females.

*Aedeagus* (Fig. 4) apically expanded and narrowed in anterior third. Ventrally, lateral margins showing strong protuberances in basal third. Endophallic orifice visible in the basal third of the aedeagus. Preputial sac comprising two membranous lobes. Endophallic armature made up of small spines. Long and narrow parameres (Fig. 5). Scarce pores with or without setae. Two apical setae as long as paramere.

#### Biology.

On moss.

#### Etymology.

Derived from Cameroon, where the type locality of this new species is found.

### Key of the *Micrambe alluaudi* group from Africa

At the start of our investigation we were soon satisfied that a number of *M. johnstoni* (Scott) *M. helichrysi* Scott, and *M. alluaudi* (Scott) could be reliably recognised on external characters: body oval, elongated and moderately convex. Simple, short, recumbent, and whitish pubescence. Pronotum slightly transverse, sub-square, or moderately transverse. Callosities, oval, elongated (1/3 of the side length), visible from above, generally obliquely cut, not protruding from the lateral margin of the pronotum. Aedeagus apically expanded. Strong callosity in the basal third of the lateral margin. Very small, triangular parameres. Provided with three or four apical setae longer than the paramere. Paramere arms very dilated distally. Atypical forms occur, and cannot be distinguished except on parameres. Key is incorporated below:

**Table:** 

1	Tarsal formula 5-5-5 in both sexes	**2**
–	Tarsal formula 5-5-4 in males and 5-5-5 in females. Dark grey-brown; many specimens reddish grey-brown along the suture and base of the pronotum; antennae and legs yellowish grey-brown. Pronotum (Fig. 12) little transverse or sub-squared (RD= 1.4). Lateral margins parallel from the callosity to shortly after the middle; next, converging towards the base. Aedeagus (Fig. 13). Parameres (Fig. 14). Length: 2.1–2.2 mm	***johnstoni* (Scott)**
2	Uniformly dark grey-brown; in some species the base of the elytra and the pronotum side are reddish; testaceous legs and antennae; dark antennal mace	**3**
–	Variable in colour, elytra usually dark grey-brown (sometimes with a more or less yellowish grey-brown spot along the suture; pronotum yellowish grey-brown; the head may be the same colour or dark although some specimens may be entirely yellowish grey-brown or dark grey-brown. Lateral margins parallel from the callosity to the basal third and from there converging towards the base (Fig. 9). Aedeagus (Fig. 10). Parameres (Fig. 11). Length: 1.9–2.3 mm	***helichrysi* (Scott)**
3	Pronotum (Fig. 6) little transverse, sub-squared (RD= 1.3). Large callosities (1/3 of side length). Lateral margins parallel from the callosity to shortly after the middle and from there converging towards the base. Aedeagus (Fig. 7). Parameres (Fig. 8). Length: 2.3–2.4 mm	***alluaudi* (Scott)**
–	Pronotum (Figs 1, 3) little or moderately transverse (RD= 1.5). Smaller callosities (1/4 of side length). Lateral margins parallel from the callosity to the basal quarter and from there converging towards the base. Aedeagus (Fig. 4). Parameres (Fig. 5). Length: 1.7–1.9 mm	***camerunensis* sp. n.**

**Figures 6–8.**
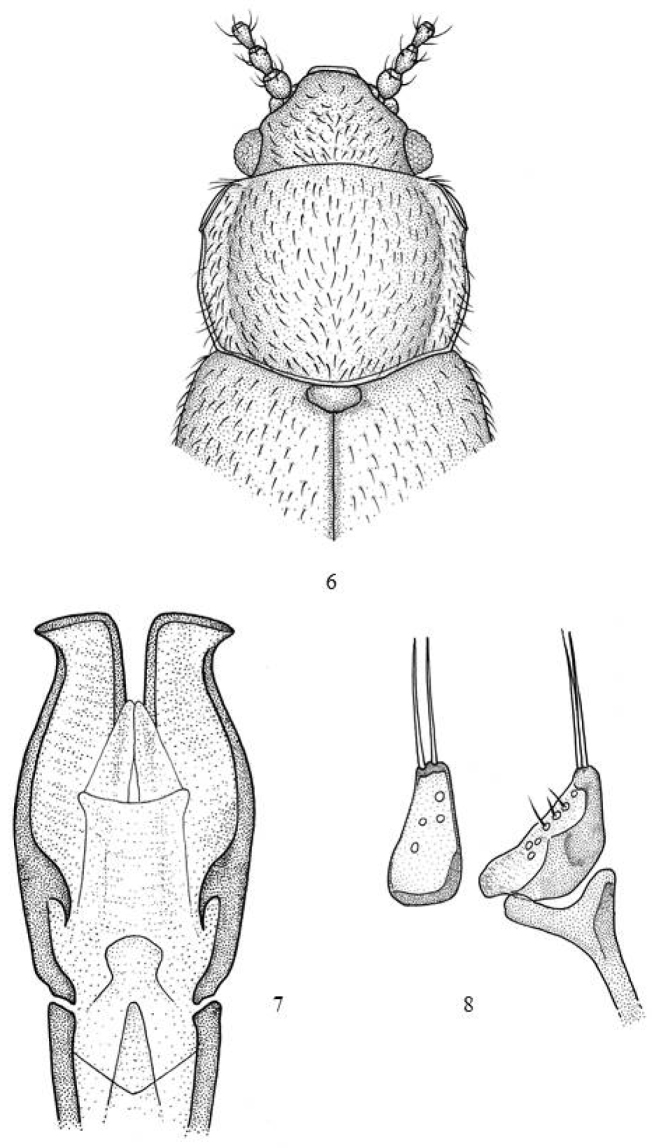
*Micrambe alluaudi*: **6** General view **7** aedeagus **8** paramere dorsal and lateral view.

**Figures 9–11.**
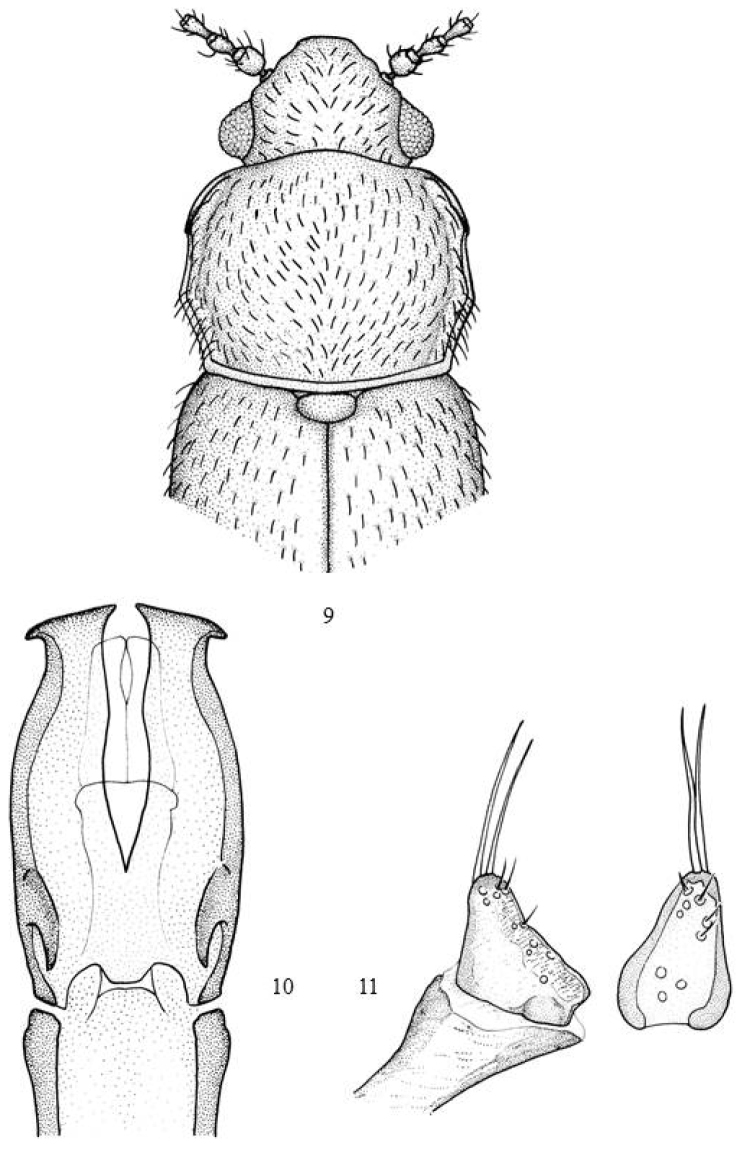
*Micrambe helichrysi*: **9** General view **10** aedeagus **11** paramere lateral and dorsal view.

**Figures 12–14.**
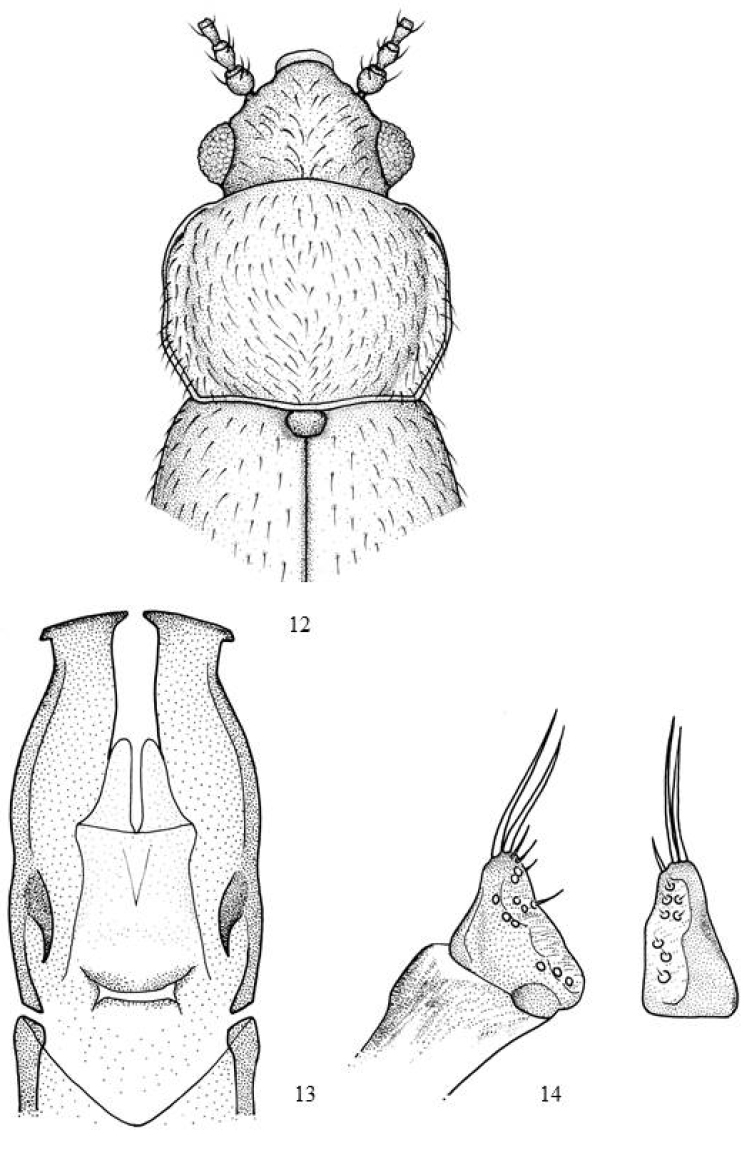
*Micrambe johnstoni*: **12** General view **13** aedeagus **14** paramere lateral and dorsal view.

## Discussion

Coombs and Woodroffe (1962) analyse the characteristics of the genus *Micrambe* from East Africa and designate the constant differences of their aedeagus with the species of paleartic dispersion. According to these authors, there is a “type of palaeartic aedeagus” that could be represented by that of *M. ulicis* (Stephens) and an African type that presents as characteristics: the apical expansion of the aedeagus present in few paleartic species and pronounced callosity in the basal third of the lateral margin. This type of aedeagus appears in the majority of the species that are distributed, throughout Eastern Africa, from Cap to Egypt. A third type, is present in a few species and could be represented by *M. alluaudi* (Scott).

In Africa there is a significant fauna rich in species of *Micrambe*, that is distributed from Egypt to Cap (South Africa). This extensive mountainous region, throughout East Africa, and like other families of Coleoptera (Jeannel 1942), could be populated by native species, from the two extremes, austral and paleartic, and the orophiles. During the quaternary period the continuity of the mountain chains broke. After the decline of volcanic activity and erosion, the mountain ranges became true islands of alpine climate, isolated in the midst of the tropical climate oceans (Jeanel 1942). In these mountains, a small number of species from the south they developed in a cold climate and have therefore been relegated to the high mountains (Rwenzori, Mount Elgon, Kenya, and Kilimanjaro) in the equatorial zone, between 3,300 m and 4,400 m, thus showing a discontinuous distribution. Their habitat is restricted exclusively to different species of Lobelia sp., Senecio sp., and Helycrysum sp. (Bruce 1952, 1960; Grouvelle 1908; Scott 1935) in which they cohabit with other coleopteran species. The forms that populate it in general are brachythera forms, bicolor or uniformly dark. They have a very small parameres, not flattened (Fig. 5) and subject to the aedeagus by arms distally very dilated (Fig. 4).

The group of thermophilic species, possibly derived from eastern lines, are distributed by and from this mountain chain towards the west, by the great equatorial forests and, therefore, have a continuous distribution. Among them, *Micrambe camerunensis*, species that shows an external morphology similar to *M. alluaudi* and that it is only possible to differentiate it by the configuration of the male genital apparatus.

## Supplementary Material

XML Treatment for Micrambe camerunensis
